# Supplementary figures and images for: Comprehensive analysis of the circadian nuclear and cytoplasmic transcriptome in mouse liver
Source: PLoS Genet. 2022 Aug 3;18(8):e1009903. doi: 10.1371/journal.pgen.1009903 (PMC9377612; doi:10.1371/journal.pgen.1009903)

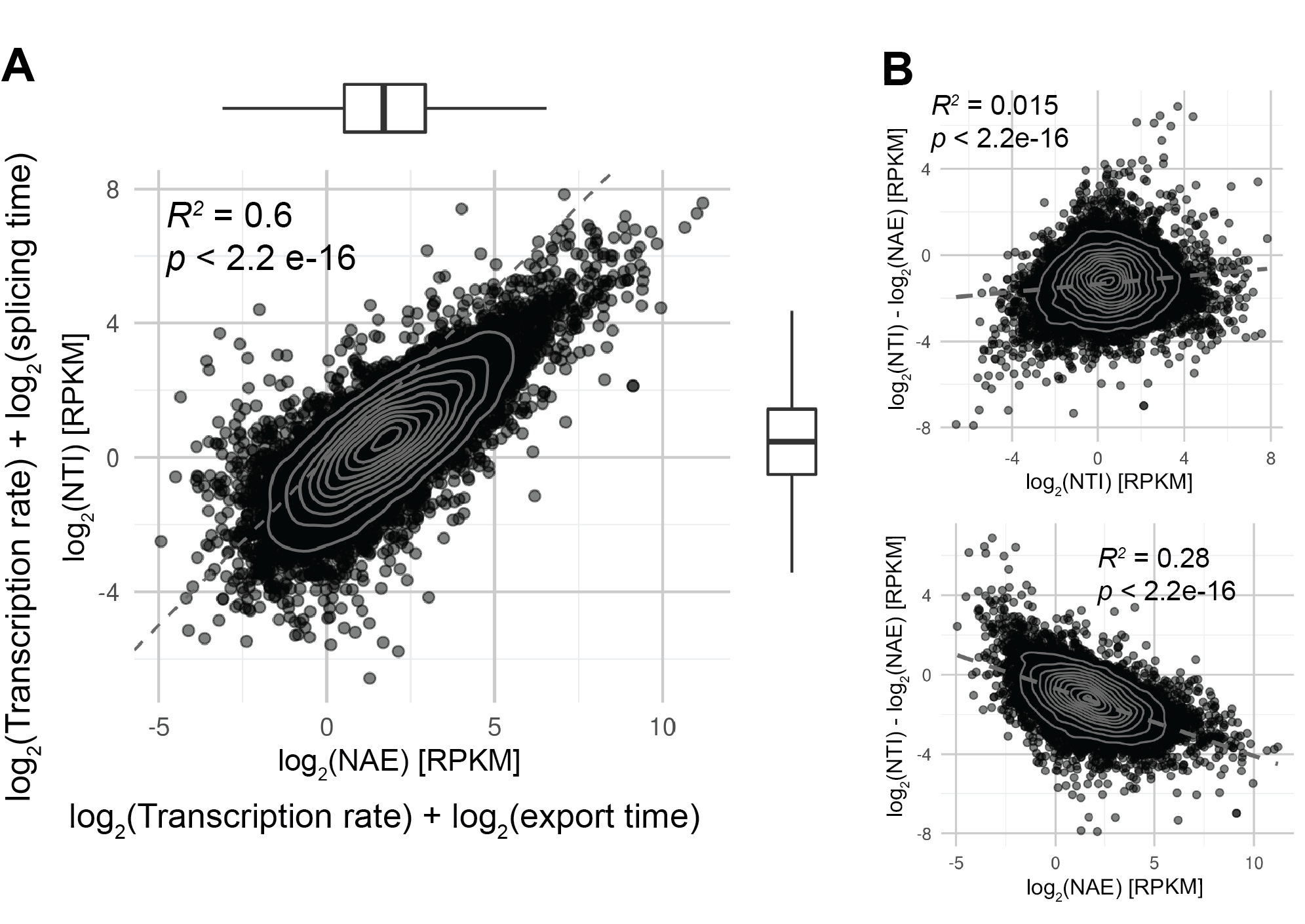

Supplement: S1 Fig — A: NTI versus NAE, in log2(RPKM), averaged over 6 time points. According to our model, log2(NTI) is defined as log2(Transcription rate) + log2(pre-mRNA processing time (splicing and polyadenylation)) and log2(NAE) is defined as log2(Transcription rate) + log2(export time). In grey: kernel 2D density. Boxplots on top and on the right show the distribution of NTI and NAE. B: NTI / NAE ratio in log2-scale, defined as log2(splicing time)—log2(export time), against the average expression of NTI (up) or against NAE (bottom). Grey line indicates linear regression. (TIF) [file pgen.1009903.s001.tif]

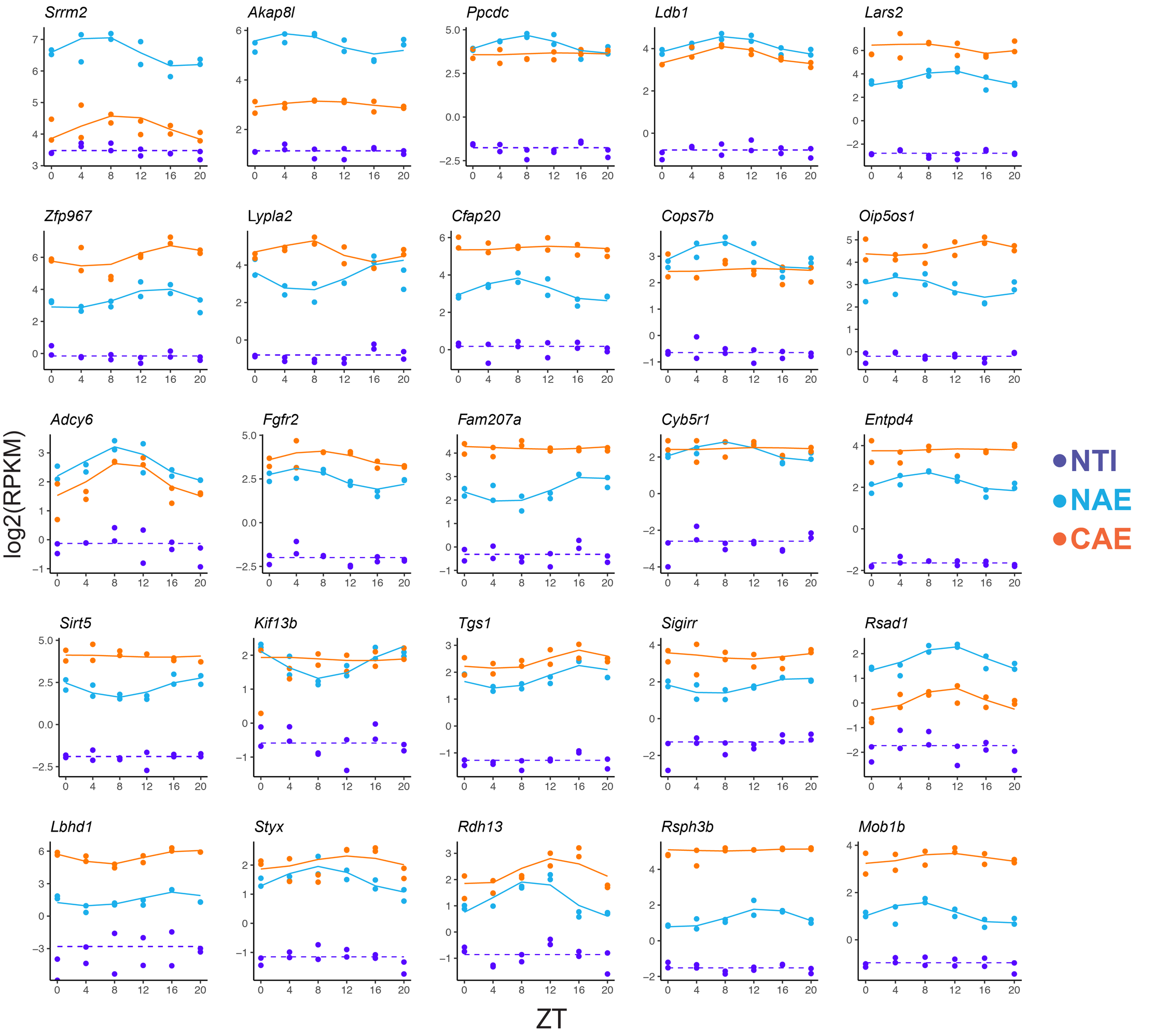

Supplement: S2 Fig — 25 profiles of genes classified as M3 (constant transcription, rhythmic export) according to the following criteria: all the genes have a log2FC > 0.8, and all classified as M2 or M4 in the analysis of step 2 (NAE vs CAE). Plots are arranged by the expression level of NAE (highest NAE Srrm2 in the upper left corner). Light blue dots and solid line: NAE, purple dots and dotted line: NTI. (TIF) [file pgen.1009903.s002.tif]
