# Supplementary material for: Comprehensive analysis of the circadian nuclear and cytoplasmic transcriptome in mouse liver
Source: PLoS Genet. 2022 Aug 3;18(8):e1009903. doi: 10.1371/journal.pgen.1009903 (PMC9377612; doi:10.1371/journal.pgen.1009903)
Supplement: Attachment — Submitted filename: Response to reviewers.docx [file pgen.1009903.s008.docx]

Reviewer's Responses to Questions

**Comments to the Authors:**

**Please note here if the review is uploaded as an attachment.**

Reviewer #1: Many genes oscillate in a time of the day dependent manner – being regulated either directly through the cells circadian clock or through systemic signals such as body temperature cycles. While transcriptional regulation is classically considered the main source of oscillating gene expression, an important contribution of post-transcriptional mechanisms is likely. In the present manuscript the authors use RNAseq data of circadian liver samples thereby comparing nuclear and cytoplasmic PolyA+ and Ribo- libraries. The combination of these libraries allows the estimation of nuclear export and cytoplasmic degradation rates, and reveals many oscillating genes with rhythmic changes in these parameters. Overall the study is well designed and includes important control experiments.

I have a few minor points:

**1) Page #5: The text says PC2 accounts for 17% of the variability (Figure: 14.7)**

Thank you for noticing this typo, which has been corrected in the main text.

**2) The authors sequenced polyA+ and Ribo- also of the cytoplasmic RNA. Are there considerable differences between rhythmic genes found in polyA+ vs Ribo- for the cytoplasm?**

In this manuscript, we do not present the temporal analysis of Ribo- cytoplasmic RNA. The reason is that it is less relevant for our model aimed at studying nuclear export, which requires comparing the temporal profiles of PolyA+ RNA in the nucleus and in the cytoplasm. The temporal data of Ribo- cytoplasmic RNA is only used as an internal control. To do so, we assessed the rhythmicity of individual genes using harmonic regression in DESeq2 [(Love, Huber, and Anders 2014)](https://www.zotero.org/google-docs/?Hv8ZYz). Specifically we compared a null model (with an intercept only) to a rhythmic model defined as a cosine with a phase and amplitude (see Table S2). We observed a strong correlation between the estimated phases and amplitudes of rhythmic cytoplasmic genes from CAE and CTE (n = 390, adjusted p-value < 0.01 for rhythmicity in both CAE and CTE, see Figure R1 below). We found a higher number of rhythmic genes in the Ribo- Cytoplasmic population (1156) compared to the polyA+ (509). This difference could be partially due to technical differences between the two sequencing methods. Libraries prepared from PolyA tail pulldown are more sensitive to the quality of RNA than those prepared from rRNA depletion [(Zhao et al. 2014)](https://www.zotero.org/google-docs/?cUfQaL), resulting in noisier quantification, which could mask some low amplitude rhythms.

Figure R1: Comparison of the temporal profiles of cytoplasmic Ribo- mRNA (CTE) and cytoplasmic PolyA+ mRNA (CAE). Left: Correlation of phases of CTE and CAE (Circular Pearson’s correlation). Right: Correlation of log_2_(Amplitude) of CTE and CAE.

**3) The PCA blot in Figure 1C shows a very nice clustering of the different libraries. Does this contain all timepoints and could the timepoints be labeled?**

Yes, all the time points are shown in Figure 1C. We did not label all the time points for the sake of readability due to the high overlap of the data point, however, we have clarified the caption.

**4) Page #9: Is Gck with a negative log2 of -1.2 nuclear enriched? What is the cut-off?**

The notion of ‘nuclear enriched’ has no unique definition. Instead of setting a cut-off on the log2-ratio of NAE and CAE, we identified transcripts that stand out in the population as being either nuclear or cytoplasmic. Statistical analysis for enrichment of protein coding genes were performed specifically for Fig 2.F (gene set enrichment analysis, see Methods and Table S3) using DESeq2, and *Gck* is in the top 30% most nuclear genes among protein coding genes. In the main text, as illustrative examples, we gave the log_2_-ratio of protein coding genes that have been previously shown to be enriched in the nucleus such as *Mlxipl, Gcgr,* and *Nlrp6* [(Bahar Halpern et al. 2015)](https://www.zotero.org/google-docs/?54YNms). However, we noticed a mistake from our side, as *Gck* was mentioned in [(Bahar Halpern et al. 2015)](https://www.zotero.org/google-docs/?tlBhtH) to be nuclear-enriched in the MIN6 cells, and not in the mouse liver. Even if *Gck* is enriched in the nucleus relative to the behavior of all genes, we remove it from the main text to avoid confusion.

**5) In the text the authors use r values while the figures depict R² values. Could they decide for one?**

Thank you for this feedback. We modified the main text to be consistent with the figures, and only show the R^2^ value, as the direction of the correlation is visible in the scatterplot.

**6) All linear regression fits show an identical p Value of < 2.2e-16. Even if almost no correlation is visible. Is this still correct (due to the high n number?)**

Yes, it is correct. Due to the large number of data points, we overlay the 2D density plot on the scatterplot in order to make the trend of the correlation more readable. As for the p-value, the function used to compute it sets by default the lowest value to 2.2 e-16, and we thus kept it in the figure.

**7) Page #15: In Fig 4D…. Is this not 4F?**

Thank you for noticing this typo, it has been corrected in the main text.

**8) Fig. 4B: Why is in model 2 the pre-mRNA higher expressed then the mRNA?**

Fig 4B is a schematic depicting the behavior of pre-mRNA and mRNA in each of the 4 models, based on a simulation with arbitrary initial conditions and processing rates. We agree that the initial scheme was confusing for the reader, and have therefore updated Fig 4B.

**9) In Figure 5 the authors compare rhythmic nuclear RNAs. Based on panel E these also show rhythms in the cytoplasm. Is this with the same acrophase?**

Rhythmic patterns of nuclear mRNA propagate to the cytoplasm, where amplitude and phase depend on the half-life of the cytoplasmic transcript. In panel E, we showed that rhythms generated by rhythmic export (genes classified as M3 in step1) were sufficiently large to be then robustly detected in the cytoplasm. The evaluation of the phases of these transcripts in the cytoplasm did not reveal any noteworthy characteristics to be discussed in this study. However, based on the reviewer's comment and in order to give a more complete view of the results, we now added an extra panel (F) showing the distribution of phases of cytoplasmic mRNA, according to their classification in step 1.

Fig R2: New panel added to Fig 5 (Panel F): Distribution of phases in the cytoplasm of genes classified as M2, M3 or M4 at step 1.

**10) Based on 4D many NAE rhythmic transcripts show a low amplitude. Could the authors comment how reliably these can be predicted (especially with n=2 I would estimate some false positive genes).**

We assume that the reviewer was referring to Figure 5D, where we model the temporal patterns of NAE and NTI, to identify transcripts that are either rhythmically transcribed and exported at a fixed rate (M2), constitutively transcribed and exported rhythmically (M3), or transcribed and exported rhythmically (M4). This modeling is combined with a model selection approach, where we probabilistically classify a gene as M2, M3, or M4, based on the measurement of two conditions (here NTI and NAE).

It is important to note that such an approach is different from standard rhythmicity analysis for one single condition and should therefore not be directly compared. As we described in [(Weger et al. 2021)](https://www.zotero.org/google-docs/?IESeF8), our approach is in essence a model-based classification scheme into different models (M1-M4). This also signifies that the statistical output is different: this method returns a probability to belong to any of the four models (Schwartz weights). Only genes classified with a probability > 0.6 were used in our analysis.

Also, fitting an explicit dynamical model (production-degradation kinetics) to the time traces also allows us to infer the model parameters (and their coefficients of variation) such as the amplitude, phase, or transcript half-life (see Methods). All these parameter estimates are reported in Table S4.

Here, we have sampled every 4 h during two nonconsecutive days under LD conditions. This study design fulfills the conditions established in [(Hughes et al. 2017)](https://www.zotero.org/google-docs/?w8JcTA) authored by a consortium of leading circadian researchers and is similar to the vast majority of the studies in the field In a recent paper, where we have established this model selection methodology (DryR), we have carefully benchmarked *dryR* using simulated count data using the described sampling frequency (4h / 2 days).

Therefore, even though this is not the focus of this paper, we have now added ‘classic’ p-values for all NAE transcripts in Table S2 (See method, harmonic regression with DESeq2). For completeness, we have also provided those p-values and the parameters of the fit for all conditions (Table S2).

**11) The authors mention a spike in rat RNA. Could they comment why this was not used for normalization. Would this result in comparable results?**

Thank you for asking the question. Initially, we had envisaged using the spikes to account for a possible “global” rhythm in the total number of mRNAs per cell, as previously reported in [(Sinturel et al. 2017)](https://www.zotero.org/google-docs/?4u2nla). Such rhythms would normally escape detection in a standard experiment that uses a fixed amount of RNA mass for library preparation. We therefore added a fixed amount of cytoplasmic and nuclear extracts from a sample of rat liver (account for 5% of the mass of sampled mouse liver). However, we underestimated the technical variability in the yield during the fractionation process, and temporal profiles were noisy after normalizing the data relative to the rat liver spike-in. For example, even the rhythms of core clock genes were aberrant. We therefore decided to proceed with a classic normalization procedure.

Reviewer #2: In the manuscript entitled “Comprehensive analysis of the circadian nuclear and cytoplasmic transcriptome in mouse liver”, Hurni et al. generate and analyze new circadian datasets from cellular fractions obtained from mouse liver and determined the kinetics of gene expression. Briefly, the authors extract RNA from nuclear and cytoplasmic fractions of mouse livers collected at different times of the day (every 4 hours) and use it to generate polyA and total RNAseq libraries which they sequence. By plugging the data into several mathematical models, the authors can determine not only genes displaying rhythmic transcription and steady state levels (as done previously by many including the Naef lab) but also degradation, splicing and nuclear export rates. Among the many findings the authors uncover a role for nuclear export in generating and modulating circadian gene expression.

The present manuscript presents both very interesting datasets as well as findings regarding not only circadian gene expression but also how gene expression in general can be modulated at the different levels. While some of the findings might seem only confirmatory, the present study is solid and constitute a body of work that should be published and shared with the scientific community. So, I strongly support publication in PLoS Genetics with minor revisions.

We thank reviewer #2 for her/his positive assessment of our work.

Minor Concerns

**- Most of the results are based on the analysis of RNAseq datasets. In this context it would be good if the authors could speculate how the oscillations of the genes in the M4 group is generated.**

Mounting evidence supports the role of RNA regulatory processes in controlling circadian rhythms, both during transcription (co-transcriptional splicing, recruitment of export machinery, and specific RNA-Binding proteins), and post-transcriptionally (RNA-editing, nuclear retention and nuclear export, regulation cytoplasmic stability and translation efficiency). Our dataset allows us to uncover rhythmic patterns that are modulated at post-transcriptional levels, more specifically the export from the nucleus to the cytoplasm (M3 and M4 genes at step 1) and the cytoplasmic mRNA stability (M3 and M4 genes at step 2). Oscillations in group M4 combine both rhythmic production and removal, which can lead to more flexible amplitude and phase relationships compared to models M2 and M3 [(Wang et al. 2018)](https://www.zotero.org/google-docs/?9K3Z2a)**.**

Regarding possible mechanisms, for step 2, fluctuation in mRNA stability can occur through the rhythmic activity of RNA Binding Proteins [(Benegiamo, Brown, and Panda 2016)](https://www.zotero.org/google-docs/?Tg1rJM), by varying the Poly(A) tail length [(Kojima, Sher-Chen, and Green 2012)](https://www.zotero.org/google-docs/?5Z4lTZ), or by targeted miRNA [(Du et al. 2014)](https://www.zotero.org/google-docs/?o2QVTO). Less is known for rhythmic regulation at step 1. RNA processing factors and nuclear bodies, such as nuclear paraspeckles, could potentially be involved in regulating nuclear mRNA retention in the circadian context. We now extended the Discussion section about rhythmic post-transcriptional regulation both at step1 and step 2.

**- The fact that splicing seems less efficient in longer genes is no surprising. Maybe the authors could interpret this in the context of effects of transcriptional elongation on cotranscriptional splicing.**

Our statement in Fig. 3 was that short genes tend to be more post-transcriptionally spliced, which is equivalent to the statement that short genes are less efficiently spliced co-transcriptionally. We have now clarified this in the main text.

Reviewer #3: Hurni et al. produced time series RNA-seq data sets from fractionated mouse liver cells and used the obtained RNA measurements to build a mathematical model, which allows to investigate RNA processing steps namely nuclear export and cytoplasmic degradation. Furthermore, they also studied the proportion of co-transcriptionally and post-transcriptionally spliced genes through the intronic signals present within nuclear total pre-mRNA and the nuclear polyadenylated pre-mRNA and reported a strong correlation between gene length (using only protein coding transcripts) and the tendency towards co-transcriptional or post-transcriptional splicing.

Overall, I found the idea driving this work to be interesting. Valuable data sets were produced, which will be appreciated by others investigating circadian regulation underlying RNA processing steps. Potentially interesting results, though still preliminary, are presented. I have a few major and minor concerns which should be addressed before publication.

We thank this reviewer for his/her insightful comments and suggestions. Note that the comments pertain largely to the regulation of alternative splicing, which is only a minor aspect of our work. Instead, by fractionating nuclear and cytoplasmic RNA pools, we designed this study to investigate, for the first time, the role of nuclear export rate in circadian nuclear and cytoplasmic mRNA (spliced) accumulation. While we did use Kallisto [(Bray et al. 2016)](https://www.zotero.org/google-docs/?tRfhoI) to estimate transcript specific expression levels and in particular prune non-expressed transcripts, obtaining reliable analyses at the transcript level is not yet realistic due to the limitation of short read technology. We have therefore summarized our analysis at the gene level, or grouped transcripts by biotype to distinguish some broad classes. An isoform specific description might become realistic in the future with the advances in longer read sequencing.

Major concerns:

**1- Overall, I found that the main conclusions are often hidden in a description of the methodology. This is often the case in the results section where the authors frequently describe the methods, but not the interpretation of the results presented.**

Thank you for this comment, we have now reduced the amount of methodological statements in the main text and tried our best to emphasize the main conclusions better throughout the manuscript.

**2- In the discussion a summary of the results is presented, but little is mentioned regarding the contextualization of these results within the landscape of published data. This is particularly evident when referring to the splicing findings. The literature on circadian regulation of splicing seems to have been, to a large extend, neglected (e.g. McGlincy NJ, et al. Genome Biol. 2012. doi: 10.1186/gb-2012-13-6-r54;. Benegiamo, G. et al RNA Process; Springer, 2016; pp. 107–122.; El-Athman R, et al. EBioMedicine. 2018. doi: 10.1016/j.ebiom.2018.06.012; Torres M, et al. Wiley Interdiscip Rev RNA. 2018 doi: 10.1002/wrna.1467).**

Thanks for this comment. Like we explained in the general reply to this reviewer above, the novelty of the present study was to fractionate samples in order to identify rhythms in mRNA abundance that originate from regulated nuclear export or cytoplasmic half-lives. Thus, while circadian regulation of alternative splicing is currently of great interest, we investigated the rhythmic patterns of the mentioned RNA fractions at the gene level only. This choice, not to look into the transcript isoforms, was to keep the already complex analysis manageable. Indeed, stratifying our results on nuclear and cytoplasmic half-lives and on rhythmic post-transcriptional regulation would make the analysis too complex, especially since transcript specific quantifications remain challenging with short read sequencing.

Nevertheless, we agree that a better discussion of the literature on circadian alternative splicing was necessary and have now included the discussion of the listed references.

**3- How much of the findings apply only to the liver (which is of course fine and to some degree expected)? Alternative splicing is known to show tissue specificity (Baralle, F et al. Nat. Rev. Mol. Cell Biol. 2017) and also circadian regulation seems to be tissue specific (Litovchenko M, et al. Sci Adv. 2021 Jan 29;7(5):eabc3781. doi: 10.1126/sciadv.abc3781.; Harder L, et al. Bioessays. 2020 May;42(5):e1900158. doi: 10.1002/bies.201900158.). Can the authors use published mammalian circadian data sets to verify some of their conclusion, or to compare the outcomes using different tissues?**

We have made extensive searches but have not found similar circadian datasets of purified nuclear and cytoplasmic fractions in other mammalian tissues. Therefore we cannot make comparisons on the origins of circadian mRNA accumulation (e.g. Models 3 and 4 vs Model 2) in other tissues.

Though this was not the focus of the present work, several datasets of cellular fractionation without the temporal dimension are available. We thus compared our findings about splicing (on temporally averaged expression level), and more specifically, on the dependency of gene length with co- or post-transcriptional splicing. We looked at a published dataset of fractionated mESCs [(Yeom et al. 2021)](https://www.zotero.org/google-docs/?CiGX3c), where the authors computed the splicing efficiency. We found a correlation between the gene length and the splicing efficiency, such that long genes tended to be more co-transcriptionally spliced, similarly to our results (Pearson’s correlation between gene length and “Fraction inclusion” of -0.31). In the revised version of the discussion, we have therefore extended the discussion about splicing accordingly.

**Figure R3**. Correlation between the Fraction of intron Inclusion (FI) and intron length in mESCs (data from Yeom et al. 2021). Low FI values indicate efficient co-transcriptional splicing. Pearson’s correlation: 0.4 (not in the manuscript, discussed in the discussion session).

**4- Related to the previous comment: it is relevant to specify, for the different conclusion, that the current findings apply only to liver tissues. I assume that the RNAseq data is submitted to a public repository, however this is not indicated nor is an accession number provide, which could be used to retrieve the data, after publication of the manuscript**.

We updated the discussion section such that it is clear that our current findings only apply to the mouse liver.

All the RNA-seq raw data and processed data were deposited on GEO (GSE182856), however, by the time of the submission, these data were not made public. Due to an omission, the tables containing the processed data and the classification were missing in the submission to PLoS Genetics, which we have now corrected. Unfortunately we omitted to provide the token for the referees in the initial submission. Here is now the token: “udodiyuqbryrlqx”.

**5- Some references are missing, for e.g. the authors write “…to couple circadian genes and physiological pathways.”, pg 3, a reference would be needed.**

Thank you for this observation, we added relevant citations for the readers [(Asher and Schibler 2011; Eckel-Mahan and Sassone-Corsi 2013)](https://www.zotero.org/google-docs/?yEBlGy).

**6- The authors present a circadian analysis based on the expression of the genes, yet often the individual transcripts oscillate, but when summarizing their expression, the result is a non-oscillating gene (expression), this of course will influence the conclusions and should be taken into account, in particular considering the underlying splicing analysis**.

As we explained above, the inference of alternative transcript isoforms levels from short read sequencing data remains challenging, and it was not the aim of this study to perform the model selection analysis on the level of individual transcript isoforms, as this requires accurate quantification of the transcript levels. We agree with the reviewer that it is possible that some rhythms at the transcript isoform level may be masked. In that sense our analysis can be considered as conservative, but we would prefer to wait for the availability of long read transcript quantification for the next steps. Note that as a first step in this direction, we grouped individual transcripts by relevant biotype for the steady-state analyses. The rationale behind this was that different biotypes are not processed the same way [(Palazzo and Lee 2018)](https://www.zotero.org/google-docs/?D6qRaG). We have now mentioned in the Results and Discussion that long read sequencing will be promising in the hopefully near future for refined isoform-levels dynamic transcriptome analyses.

**7- It is often not clear which isoform of a certain gene is being considered and several of the genes mentioned have more than one isoform.**

To address this we are now providing new supplementary tables (S1 and S2) containing the list of isoforms expressed for each gene. Whenever the expression is summarized at the gene-level or grouped by similar biotype, the isoforms used for the quantification are listed.

**8- How would the authors account for alternative splicing using their model, or are they considering simply one transcript per pre-RNA, and if yes, what would be the rationale to ignore alternative splicing events?**

We do not presently know how to generalize the current model to a group of alternatively spliced transcripts. This would significantly increase the number of parameters in the model, which would render the parameter inference non-identifiable. This is why we performed the analysis of rhythm and estimation of half-lives at the whole-gene level only. The fact that different isoforms may have different rhythmic patterns that would be hidden at the gene-level is now clearly mentioned in the discussion, and we referenced relevant papers [(El-Athman et al. 2019)](https://www.zotero.org/google-docs/?3lkEk1).

**9- The authors refer to “splicing time” how is splicing time determined and how was it validated?**

In our set of differential equations describing the lifecycle of an RNA transcript, the nuclear pre-mRNA (NTI) is transformed into mRNA (NAE) through a splicing rate *s*, which is fitted from the data. Thus, splicing time, defined as $\frac{log(2)}{s}$, directly derives from our model. We acknowledge that this is a very naive view of the splicing process, and we discuss this issue in the discussion section. Note that the splicing time is identifiable only for some of the models and that we have set a prior on it (Methods), allowing the splicing time to range from x to y. These points have been clarified in the discussion section, as also suggested under points 2 and 3 from this reviewer.

**10- The authors refer to Correlation coefficient (r) values in the results section, I could not find a description for the type of correlation computed. Overall the information regarding statistics is lacking throughout the manuscript. The authors show (e.g. Fig 4) potentially oscillating genes, but do not provide p-values/adjusted p-values for the significance of such oscillation.**

Thank you for this comment (see also our replies to Referee 1, point x). In Figure 4 we jointly model the temporal patterns of NAE and CAE, to identify transcripts that are either rhythmically transcribed and degraded in the cytoplasm at a fixed rate (M2), constitutively transcribed and degraded rhythmically (M3), or transcribed and degraded rhythmically (M4). This modeling is combined with a model selection approach, where we probabilistically classify a gene as M2, M3, or M4, based on the measurement of two conditions (here NAE and CAE). As such, it is important to note that this approach is very different from standard rhythmicity analysis for one single condition and should therefore not be directly compared. For example for M2 genes with long half lives, it is expected that the rhythms in CAE will be low amplitude (Fig. 4F M2). It is thus expected that many genes classified as M2 will show low statistical significance in their CAE profile.

As we described in [(Weger et al. 2021)](https://www.zotero.org/google-docs/?QVuHxb), our approach is in essence a model-based probabilistic classification scheme into different models (M1-M4). This also signifies that the statistical output is different: this method returns a probability to belong to any of the four models (Schwartz weights). Only genes classified with a probability >0.6 were used in our analysis to avoid ambiguous classifications.

Fitting an explicit dynamical model (production-degradation kinetics) to the time traces also allows us to infer the model parameters (and their coefficients of variation) such as the amplitude, phase, or transcript half-life (see Methods). All these parameter estimates are reported in Table S4.

Therefore, even though this is not the focus of this paper, we have now added rhythmicity p-values for all transcripts in Table S2 (See method, harmonic regression with DESeq2).

**10.2 - Also in Fig 4F in some plots an error for the half-life values is provided and in some not. The information regarding what type of error this is (e.g. SD, SEM) is missing, as well as which N was used.**

Fig 4F shows representative temporal profiles of genes classified as M2, M3 and M4. In M3 and M4, half-lives have circadian variations because of rhythmic degradation. The mean half-life with the corresponding min/max are indicated on the plot. The explanation on the indicated values was indeed missing in the caption, and we have now completed it. In the figure, in order to keep it readable, we did not add the error, however, the SEM of the estimated parameters are provided in Supplementary Table S4.

**11- In Fig 6 D, F it is not clear how many nuclei were used for the quantification, statistics are also missing to evaluate whether the apparent differences observed are significant.**

Thank you for pointing out this missing information, we updated the caption to include statistical tests (pairwise t-test with Bonferroni correction) to compare the nuclear-to-cytoplasmic ratio, and additional information about the smFISH images quantification.

**12- Which splicing factors are the authors considering for their analysis? For e.g. in Fig 2 the authors refer to “splicing (SR family members)”, but this would be a rather reductionist view of the group of splicing factors, it should be made clear in the manuscript which SFs are being considered.**

We agree that this was confusing and have therefore remade Figure 2D. The idea was to highlight illustrative genes which were significant in a GO analysis of nuclear enriched genes (now provided as Table S3), and not to study the behavior of splicing factors exhaustively. Two of those categories (GO:0043484, regulation of RNA splicing, and GO:0006376, mRNA splice site selection) were related to splicing, as we now clearly state in the caption.

**13- The authors mentioned changes in the circadian profile of splicing factors such as Hnrnph1 or Srsf1, where alterations in spliceosome complex elements such as U2af1 detected?**

In the nucleus (step 1), *U2af1* is classified as M2 (rhythmic transcription, constant export), peaking around ZT20. The amplitude of the nuclear mRNA is however low (log_2_(FC) of 0.7), and subsequently, the remaining amplitude in the cytoplasm was not detected by our model. We compared these fits using an alternative method (harmonic regression with DESeq2, Table S2): *U2af1* in the nucleus (both NTI and NAE) are again rhythmic with a p-value < 0.05. CAE is considered again as arrhythmic in the cytoplasm, note however the p-value of 0.06. *U2af1* is thus a gene where it is difficult to robustly distinguish low amplitude rhythms from noise.

**14- In the results section, the authors mentioned that “The splicing regime is strongly influenced by the gene length, or more precisely by the relation of splicing and elongation time”. However, the authors have only focused on the transcripts with protein coding biotypes during this analysis, thus it should be specified that this conclusion is only valid for such transcripts.**

We indeed restricted our analysis on protein coding genes only, and we modified the text of the results and the discussion sections to clarify it.

**15- What was the rationale to focus on PARBZip family members?**

PARbZip transcription factors are among the most prominent direct clock output genes. Additionally, they bind core circadian clock genes through D-boxes. Still, the role of PARbZip transcription factors in the circadian clock is debated. Typically, KO animal models do not show alterations of their circadian locomotor activity, despite the alteration of the rhythmicity of hundreds of genes [(Weger et al. 2021)](https://www.zotero.org/google-docs/?JDe2VI). Given that PARbZIP transcription factors, and particularly *Dbp*, are among the genes with the highest amplitudes in the liver, and their close link with the circadian core clock, we included them in our analysis.

**16- Have the authors found a correlation between the number of transcripts (or maybe number of circadian transcripts) from a given gene and co- vs post-transcriptional splicing?**

Thank you for this interesting suggestion. Unfortunately in our dataset, at the genome-wide level, we did not find a correlation between the number of expressed isoforms and the log_2_(NTI/NAI) ratio, which we use as a proxy for co- vs post-transcriptional splicing in Fig 3 (Pearson’s correlation = 0.007, p-value = 0.5).

**Minor concerns:**

• In the Methods section, the catalogue numbers, company names, and locations are missing.

• Figure 1, panel E: It is currently difficult to differentiate between dashed and solid lines (exon and introns).

• There are few typos in the manuscript e.g. “bioype”, “focussing”.

• The magnification details for the microscopy pictures is missing (Figure 6).

• Missing axis labels in Figure 6A, Figure S2.

• In the Microscopy and image analysis, missing “analysis” in the sentence “the image was performed using ImageJ”.

• Missing details on ImageJ and microscope software version.

We thank the reviewer for her/his careful reading, and we have now fixed those errors.

Overall, the study design and purpose are interesting, but a better description and interpretation of the results is needed to highlight the major outcomes, as well as improving the contextualization and association of ideas in the discussion.

**Supplementary references:**

[Asher, Gad, and Ueli Schibler. 2011. “Crosstalk between Components of Circadian and Metabolic Cycles in Mammals.” *Cell Metabolism* 13 (2): 125–37. https://doi.org/10.1016/j.cmet.2011.01.006.](https://www.zotero.org/google-docs/?rZxwNe)

[Bahar Halpern, Keren, Inbal Caspi, Doron Lemze, Maayan Levy, Shanie Landen, Eran Elinav, Igor Ulitsky, and Shalev Itzkovitz. 2015. “Nuclear Retention of MRNA in Mammalian Tissues.” *Cell Reports* 13 (12): 2653–62. https://doi.org/10.1016/j.celrep.2015.11.036.](https://www.zotero.org/google-docs/?rZxwNe)

[Benegiamo, Giorgia, Steven A. Brown, and Satchidananda Panda. 2016. “RNA Dynamics in the Control of Circadian Rhythm.” In *RNA Processing: Disease and Genome-Wide Probing*, edited by Gene W. Yeo, 107–22. Advances in Experimental Medicine and Biology. Cham: Springer International Publishing. https://doi.org/10.1007/978-3-319-29073-7_5.](https://www.zotero.org/google-docs/?rZxwNe)

[Bray, Nicolas L., Harold Pimentel, Páll Melsted, and Lior Pachter. 2016. “Near-Optimal Probabilistic RNA-Seq Quantification.” *Nature Biotechnology* 34 (5): 525–27. https://doi.org/10.1038/nbt.3519.](https://www.zotero.org/google-docs/?rZxwNe)

[Du, Ngoc-Hien, Alaaddin Bulak Arpat, Mara De Matos, and David Gatfield. 2014. “MicroRNAs Shape Circadian Hepatic Gene Expression on a Transcriptome-Wide Scale.” Edited by Louis Ptáček. *ELife* 3 (May): e02510. https://doi.org/10.7554/eLife.02510.](https://www.zotero.org/google-docs/?rZxwNe)

[Eckel-Mahan, Kristin, and Paolo Sassone-Corsi. 2013. “Metabolism and the Circadian Clock Converge.” *Physiological Reviews* 93 (1): 107–35. https://doi.org/10.1152/physrev.00016.2012.](https://www.zotero.org/google-docs/?rZxwNe)

[El-Athman, Rukeia, Dora Knezevic, Luise Fuhr, and Angela Relógio. 2019. “A Computational Analysis of Alternative Splicing across Mammalian Tissues Reveals Circadian and Ultradian Rhythms in Splicing Events.” *International Journal of Molecular Sciences* 20 (16): 3977. https://doi.org/10.3390/ijms20163977.](https://www.zotero.org/google-docs/?rZxwNe)

[Hughes, Michael E., Katherine C. Abruzzi, Ravi Allada, Ron Anafi, Alaaddin Bulak Arpat, Gad Asher, Pierre Baldi, et al. 2017. “Guidelines for Genome-Scale Analysis of Biological Rhythms.” *Journal of Biological Rhythms* 32 (5): 380–93. https://doi.org/10.1177/0748730417728663.](https://www.zotero.org/google-docs/?rZxwNe)

[Kojima, Shihoko, Elaine L. Sher-Chen, and Carla B. Green. 2012. “Circadian Control of MRNA Polyadenylation Dynamics Regulates Rhythmic Protein Expression.” *Genes & Development* 26 (24): 2724–36. https://doi.org/10.1101/gad.208306.112.](https://www.zotero.org/google-docs/?rZxwNe)

[Love, Michael I., Wolfgang Huber, and Simon Anders. 2014. “Moderated Estimation of Fold Change and Dispersion for RNA-Seq Data with DESeq2.” *Genome Biology* 15 (12): 550. https://doi.org/10.1186/s13059-014-0550-8.](https://www.zotero.org/google-docs/?rZxwNe)

[Palazzo, Alexander F., and Eliza S. Lee. 2018. “Sequence Determinants for Nuclear Retention and Cytoplasmic Export of MRNAs and LncRNAs.” *Frontiers in Genetics* 0. https://doi.org/10.3389/fgene.2018.00440.](https://www.zotero.org/google-docs/?rZxwNe)

[Sinturel, Flore, Alan Gerber, Daniel Mauvoisin, Jingkui Wang, David Gatfield, Jeremy J. Stubblefield, Carla B. Green, Frédéric Gachon, and Ueli Schibler. 2017. “Diurnal Oscillations in Liver Mass and Cell Size Accompany Ribosome Assembly Cycles.” *Cell* 169 (4): 651-663.e14. https://doi.org/10.1016/j.cell.2017.04.015.](https://www.zotero.org/google-docs/?rZxwNe)

[Wang, Jingkui, Laura Symul, Jake Yeung, Cédric Gobet, Jonathan Sobel, Sarah Lück, Pål O. Westermark, Nacho Molina, and Felix Naef. 2018. “Circadian Clock-Dependent and -Independent Posttranscriptional Regulation Underlies Temporal MRNA Accumulation in Mouse Liver.” *Proceedings of the National Academy of Sciences* 115 (8): E1916–25. https://doi.org/10.1073/pnas.1715225115.](https://www.zotero.org/google-docs/?rZxwNe)

[Weger, Benjamin D., Cédric Gobet, Fabrice P. A. David, Florian Atger, Eva Martin, Nicholas E. Phillips, Aline Charpagne, Meltem Weger, Felix Naef, and Frédéric Gachon. 2021. “Systematic Analysis of Differential Rhythmic Liver Gene Expression Mediated by the Circadian Clock and Feeding Rhythms.” *Proceedings of the National Academy of Sciences* 118 (3). https://doi.org/10.1073/pnas.2015803118.](https://www.zotero.org/google-docs/?rZxwNe)

[Yeom, Kyu-Hyeon, Zhicheng Pan, Chia-Ho Lin, Han Young Lim, Wen Xiao, Yi Xing, and Douglas L. Black. 2021. “Tracking Pre-MRNA Maturation across Subcellular Compartments Identifies Developmental Gene Regulation through Intron Retention and Nuclear Anchoring.” *Genome Research*, April, gr.273904.120. https://doi.org/10.1101/gr.273904.120.](https://www.zotero.org/google-docs/?rZxwNe)

[Zhao, Wei, Xiaping He, Katherine A. Hoadley, Joel S. Parker, David Neil Hayes, and Charles M. Perou. 2014. “Comparison of RNA-Seq by Poly (A) Capture, Ribosomal RNA Depletion, and DNA Microarray for Expression Profiling.” *BMC Genomics* 15 (1): 419. https://doi.org/10.1186/1471-2164-15-419.](https://www.zotero.org/google-docs/?rZxwNe)
