# Supplementary material for: Comprehensive analysis of the circadian nuclear and cytoplasmic transcriptome in mouse liver
Source: PLoS Genet. 2022 Aug 3;18(8):e1009903. doi: 10.1371/journal.pgen.1009903 (PMC9377612; doi:10.1371/journal.pgen.1009903)
Supplement: Attachment — Submitted filename: Response to reviewers_resubmission.docx [file pgen.1009903.s009.docx]

**Responses to the review comments**

**Reviewer #3: Hurni et al. have revised the manuscript following the recommendations of the reviewers. I have carefully read the rebuttal letter and the replies to the comments of the three reviewers, which I found clear and convincing. I would also like to praise the authors for carrying out additional analysis and introducing several clarifications and additional discussion.**

**This is a very interesting work, which fully deserves publication in PLOS Genetics. I have though a few minor comments, all related to discrepancies between the rebuttal letter and the revised version of the manuscript, which the authors can easily solve.**

**I fully agree with all the replies from the authors in the rebuttal letter, however, for some of the points, I could not find, in the version of the manuscript with corrections marked, the corresponding additions/clarifications, or not as clear and as detailed as in the letter. Thus, I would ask the authors to carefully compare rebuttal vs manuscript for the points listed below where I use the same numbering as in the rebuttal letter.**

**2- In the discussion a summary of the results is presented, but little is mentioned regarding the contextualization of these results within the landscape of published data. This is particularly evident when referring to the splicing findings. The literature on circadian regulation of splicing seems to have been, to a large extend, neglected (e.g. McGlincy NJ, et al. Genome Biol. 2012. doi: 10.1186/gb-2012-13-6-r54;. Benegiamo, G. et al RNA Process; Springer, 2016; pp. 107–122.; El-Athman R, et al. EBioMedicine. 2018. doi: 10.1016/j.ebiom.2018.06.012; Torres M, et al. Wiley Interdiscip Rev RNA. 2018 doi: 10.1002/wrna.1467).**

**Reply: (…) Nevertheless, we agree that a better discussion of the literature on circadian alternative splicing was necessary and have now included the discussion of the listed references.**

There are two aspects to this point: one is that we had a formatting issue with the updates of the bibliography, while we had added and discussed these references in the main text. The update of the bibliography is now provided. Secondly, we now reorganised and expanded the discussion on alternative circadian splicing so as to make that topic better covered (page 18-19 in of the manuscript, in Discussion section).

New paragraph:

“The temporal analysis of our dataset was performed only at the whole gene-level and we therefore did not look into differential rhythmicity of splice isoforms. In previous works on circadian alternative splicing, it was found that mRNAs encoding for splicing-related genes frequently exhibited 24-h rhythmic expression with a bimodal distribution of peak phases across tissues and species, indicating that they might be controlled by the circadian clock [(El-Athman et al., 2018, 2019)](https://www.zotero.org/google-docs/?3Tdo9l). Concomitantly, rhythmic alternative splicing was found to be widespread across mammalian tissues and conserved alternative splice isoforms that peaked at opposite times of the day were reported in oncogenes and tumour suppressors [(El-Athman et al., 2019)](https://www.zotero.org/google-docs/?yAZWuZ). In the liver, it was reported that the feeding/fasting cycle modulates hepatic circadian alternative splicing in an exon-dependent manner [(McGlincy et al., 2012)](https://www.zotero.org/google-docs/?hMJ3vA). In general, there is a risk that some circadian regulation will be masked with gene-level analyses. Nevertheless, inference of alternative transcript isoforms remains challenging with short read sequencing [(Amarasinghe et al., 2020)](https://www.zotero.org/google-docs/?qTuyMM), and stratifying the analysis of the half-lives by transcript isoforms would make our dynamical models too complex. Advances in long-read sequencing technologies would allow refined isoform-levels dynamic transcriptome analysis, contributing to a better understanding in physiological or pathological conditions [(El-Athman et al., 2018)](https://www.zotero.org/google-docs/?M2cuVF)”.

**8- How would the authors account for alternative splicing using their model, or are they considering simply one transcript per pre-RNA, and if yes, what would be the rationale to ignore alternative splicing events?**

**Reply: (…) The fact that different isoforms may have different rhythmic patterns that would be hidden at the gene-level is now clearly mentioned in the discussion, and we referenced relevant papers (El-Athman et al. 2019).**

As mentioned just above, the reworked last paragraph in the revised Discussion is now more complete and adequately cites the relevant works.

**9- The authors refer to “splicing time” how is splicing time determined and how was it validated?**

We have added in the main Discussion the following paragraph:

“These differential equations models describe the lifecycle of an RNA transcript, for example the nuclear pre-mRNA (NTI) is transformed into mRNA (NAE) through an effective splicing rate that combines the splicing rate of the individual introns, and which is then exported at a given rate. These are clearly highly simplified views of the complex processes, and it also remains challenging to infer those parameters from the data fits. Notably, the splicing time is identifiable only for some of the models where we have set a prior on it, in other models (e.g. static) only a relative splicing time can be obtained (Methods). Further experimental validations would be needed to assess the accuracy of the predicted splicing time”.

**References**

[Amarasinghe, S. L., Su, S., Dong, X., Zappia, L., Ritchie, M. E., & Gouil, Q. (2020). Opportunities and challenges in long-read sequencing data analysis. *Genome Biology*, *21*(1), 30. https://doi.org/10.1186/s13059-020-1935-5](https://www.zotero.org/google-docs/?qQruQd)

[El-Athman, R., Fuhr, L., & Relógio, A. (2018). A Systems-Level Analysis Reveals Circadian Regulation of Splicing in Colorectal Cancer. *EBioMedicine*, *33*, 68–81. https://doi.org/10.1016/j.ebiom.2018.06.012](https://www.zotero.org/google-docs/?qQruQd)

[El-Athman, R., Knezevic, D., Fuhr, L., & Relógio, A. (2019). A Computational Analysis of Alternative Splicing across Mammalian Tissues Reveals Circadian and Ultradian Rhythms in Splicing Events. *International Journal of Molecular Sciences*, *20*(16), 3977. https://doi.org/10.3390/ijms20163977](https://www.zotero.org/google-docs/?qQruQd)

[McGlincy, N. J., Valomon, A., Chesham, J. E., Maywood, E. S., Hastings, M. H., & Ule, J. (2012). Regulation of alternative splicing by the circadian clock and food related cues. *Genome Biology*, *13*(6), R54. https://doi.org/10.1186/gb-2012-13-6-r54](https://www.zotero.org/google-docs/?qQruQd)
